# Editorial: Towards an emerging science of customer loyalty to retail stores: explanation, drivers, and frameworks

**DOI:** 10.3389/fpsyg.2024.1365333

**Published:** 2024-02-15

**Authors:** Arturo Z. Vasquez-Párraga, Miguel A. Sahagún, Fabio Musso

**Affiliations:** ^1^Department of Marketing, University of Texas Rio Grande Valley, Edinburg, TX, United States; ^2^High Point University, High Point, NC, United States; ^3^Carlo Bo University of Urbino, Urbino, Italy

**Keywords:** explanation of store loyalty, drivers of store loyalty, store loyalty theoretical frameworks, retail stores, customer loyalty, customer satisfaction

Understanding the factors and antecedents that generate and maintain customer loyalty to retail stores is a primary concern in consumer research as it spawned an extensive publication stream during the last 50 years. Empirical research has purposefully focused on the role of individual and institutional drivers in long-established consumer outcomes such as customer loyalty and customer satisfaction. However, a coherent explanation of why and how customer loyalty to retail stores takes place is missing. Thus, the main objective of this Research Topic is to advance research that exhibits cause-effect relationships and are systematically integrated into a body of scientific knowledge.

Four peer-evaluated empirical articles provide evidence, analysis, and discussion of plausible explanations of customer loyalty to retail stores. Retail customer loyalty is examined as a factor, a mediator, and an outcome, using established theoretical models and applications to different consumer products in various demographic contexts.

- *Loyalty in the time of COVID-19* (Cruz-Milán) appraises consumer loyalty toward tourism destinations in the context of the coronavirus disruptions and offers a review of studies from 24 journal articles that have empirically investigated the drivers of destination loyalty during the pandemic in diverse geographical settings. The factors of destination loyalty found statistically significant include satisfaction, happiness, emotional values, and destination characteristics.
- *The influence of customer trust and artificial intelligence on customer engagement and loyalty* (Chen et al.) investigates how customer trust in home-sharing hosts and platforms affects customer relationships, manifested in customer engagement and loyalty. Despite the negative moderating role of artificial intelligence, customer engagement mediates a strong relationship between customer trust and loyalty.
- *How organizational trust impacts organizational citizenship behavior: Organizational identification and employee loyalty as mediators* (Dai et al.) examines how employee's trust in the organization is one of the major factors affecting employee loyalty, which,
- in turn, can reduce turnover possibility and absenteeism, improve job performance, and promote OCB and voice behavior.
- *Examining the effect of consumer experience on co-creation and loyalty for healthy meat consumption* (Meeprom et al.) examines how customer loyalty, defined as both attitudinal and behavioral, is significantly impacted by customer co-creation, which, in turn, is positively influenced by sensory experiences and health consciousness.

Overall, these four studies significantly contribute to the discovery and explanation of customer loyalty, as a factor, a mediator, and an outcome, within the meaningful explanatory models used and in diverse consumption contexts. As these examples hope to demonstrate, further research in the same direction and with similar striving results can be pursued.

## Author contributions

AV-P: Conceptualization, Investigation, Methodology, Supervision, Writing – original draft, Writing – review & editing. MS: Conceptualization, Methodology, Writing – review & editing. FM: Investigation, Methodology, Writing – review & editing.

